# The C1q-ApoE complex: A new hallmark pathology of viral hepatitis and nonalcoholic fatty liver disease

**DOI:** 10.3389/fimmu.2022.970938

**Published:** 2022-10-06

**Authors:** Livia K. L. Habenicht, Zhihua Wang, Xi Zhang, Yuanfang Li, Carolin Mogler, Julia Slotta Huspenina, Roland M. Schmid, Christian Weber, Sarajo K. Mohanta, Zhe Ma, Changjun Yin

**Affiliations:** ^1^ Klinik und Poliklinik für Innere Medizin II, Klinikum rechts der Isar, Technical University of Munich, Munich, Germany; ^2^ Institute for Cardiovascular Prevention (IPEK), Ludwig-Maximilians-University, Munich, Germany; ^3^ Institute of Pathology, Klinikum rechts der Isar, Technical University of Munich, Munich, Germany; ^4^ Tissue biobank of the Klinikum rechts der Isar, Technical University of Munich, Munich, Germany; ^5^ DZHK (German Centre for Cardiovascular Research), partner site Munich Heart Alliance, Munich, Germany; ^6^ Department of Biochemistry, Cardiovascular Research Institute Maastricht (CARIM), Maastricht University Medical Centre, Maastricht, Netherlands; ^7^ Munich Cluster for Systems Neurology (SyNergy), Munich, Germany; ^8^ Institute of Precision Medicine, The First Affiliated Hospital of Sun Yat-sen University, Guangzhou, China

**Keywords:** C1q-ApoE complex, viral hepatitis, hepatocellular carcinoma (HCC), nonalcoholic fatty liver disease (NAFLD), classical complement cascade (CCC)

## Abstract

We recently identified a high-affinity C1q-ApoE complex in human artery atherosclerotic intima lesions and in human amyloid plaques of Alzheimer’s Disease brains defining a common pathogenetic pathway of two diverse diseases, i.e. atherosclerosis and dementia. C1q is the initiating and controlling protein of the classical complement cascade (CCC), which occupies a key role in multiple acute and chronic inflammatory tissue responses. C1q is largely produced by myeloid cells including Kupffer cells (KCs) and subsequently secreted into the circulation as an inactive preprotein. Its binding partner, Apolipoprotein E (ApoE), is produced by KCs and hepatocytes and it is also secreted into the circulation, where it regulates essential steps of lipid transport. In addition to its major source, ApoE can be produced by non-liver cells including immune cells and multiple other cells depending on local tissue contexts. To initiate the CCC cascade, C1q must be activated by molecules as varied as oxidized lipids, amyloid fibrils, and immune complexes. However, ApoE is mute towards inactive C1q but binds at high-affinity to its activated form. Specifically, our studies revealed that ApoE is a CCC-specific checkpoint inhibitor *via* the formation of the C1q-ApoE complex. We proposed that it may arise in multiple if not all CCC-associated diseases and that its presence indicates ongoing CCC activity. Here, we turned to the liver to examine C1q-ApoE complexes in human B- and C-viral hepatitis and nonalcoholic fatty liver disease (NAFLD). In addition, we used multidrug-resistance-2 gene-knockout (Mdr2-KO) mice as a model for inflammatory liver disease and hepatocellular carcinoma (HCC) pathogenesis. In normal murine and human livers, KCs were the major C1q-producing cell type while hepatocytes were the primary ApoE-forming cell type though the C1q-ApoE complex was rare or nonexistent. However, significant numbers of C1q-ApoE complexes formed in both Mdr2-KO, human viral hepatitis, and NAFLD around portal triads where immune cells had infiltrated the liver. Additionally, high numbers of C1q-ApoE complexes emerged in human livers in areas of extracellular lipid droplets across the entire liver parenchyma in NAFLD-affected patients. Thus, the C1q-ApoE complex is a new pathological hallmark of viral hepatitis B and C and NAFLD.

## Introduction

Our previous observation of C1q-ApoE complexes in atherosclerosis and Alzheimer’s Disease and the characterization of ApoE as a potent checkpoint inhibitor of the CCC (1, 2) raised an important question: does the C1q-ApoE complex represent a common universal pathology of all diseases in which the CCC is active (3, 4)? C1q-ApoE complex formation requires prior activation of inactive native C1q (1) illustrating that its detection is largely restricted to diseased tissues. The ability of ApoE to directly inhibit the CCC *in vitro* and *in vivo* at high affinity provided a molecular link between previously unrelated proteins in two major clinically significant diseases and possibly beyond. As C1q is the CCC-initiating and activity-controlling protein, these data revealed that ApoE directly affects a major pathway of innate immune responses by containing the activity of the CCC and restricting it from overactivation (3, 5). Moreover, recent studies on the CCC indicate that it is involved in adaptive immune responses in a variety of physiological and disease conditions (3). We also observed that treatment of mice with a liver-specific small interfering RNA directed against C5, i.e. a key downstream mediator of the CCC and the other two complement pathways (5, 6), attenuated atherosclerosis progression in arteries and reduced the microglia response around Alzheimer plaques in brains (1). These studies deserve attention as both C1q and ApoE have been shown to be related to multiple chronic inflammatory diseases in separate genome-wide association studies (GWAS) though the molecular mechanisms of the association of C1q and ApoE with distinct disease entities had remained unclear (7, 8).

We proposed that the formation of the C1q-ApoE complex in atherosclerosis and Alzheimer’s Disease may indicate a universal common self-regulating mechanism of CCC-dependent inflammation in a wide range of inflammation-related diseases to control CCC activity´s *via* a feedback inhibition of its initiating protein (1). Here, we begin to address this issue by directing our attention towards the liver for several reasons: viral hepatitis ranges among the top four infectious diseases worldwide despite the progress in prevention medicine using vaccines as well as anti-viral treatments (9). Furthermore, some estimates show that nonalcoholic fatty liver disease (NAFLD) affect more than a quarter of the world population with still no available therapy (10, 11). Notably, NAFLD is associated with chronic liver inflammation, connective tissue accumulation and is the leading cause of liver cirrhosis and HCC (4). Similar to atherosclerosis (12) and Alzheimer’s Disease (13), various clinically important liver diseases are associated with acute, subacute and chronic inflammation in which immune cells accumulate around portal triads (4, 14). The inflammatory component of viral hepatitis and of NAFLD is believed to be the most important driver of the reorganization of liver structure in chronic hepatitis, the subsequent emergence of liver fibrosis, the development of liver cirrhosis and the promotion of HCC (4, 15). While effective treatment regimens are available for both chronic B and C viral hepatitis (16, 17), no similar clinically applicable treatments are available for NAFLD. Indeed, obesity and its associated liver disease, i.e. NAFLD, is now viewed as a global epidemic with billions of people affected as of 2016 (15). Therefore, there is a major and urgent, increasing and unmet medical need to understand the underlying mechanisms of the detrimental mechanisms underlying NAFLD. As an experimental model for inflammatory liver disease and HCC, we used multidrug resistance protein 2 knockout (Mdr2-KO) mice. The Mdr2 gene encodes P-glycoprotein which transports phosphorylcholine across the canalicular membrane into the bile (18). In Mdr2-KO mice, the absence of phospholipids in bile damages tight junctions and basement membranes of bile ducts, leading to leakage of bile acid into the extracellular space with subsequent portal inflammation (19). Mdr2-KO mice develop cholangitis with portal inflammation peaking at 3 months of age, and mice develop HCC at 16 months of age (20).

Here, we sought to examine whether the C1q-ApoE complex develops in several forms of murine and human hepatitis and to define its localization within the liver lobule during disease progression. Of particular interest was NAFLD in human livers whose initial pathology is characterized by intracellular and extracellular lipid droplets across the entire liver lobule (14). The accumulation of intra- and extracellular lipid droplets resembles intraplaque accumulation of oxidized lipids in atherosclerosis. In this regard it is of interest to note that oxidized low density lipoproteins are strong activators of C1q, i.e. an activity which is not shared by native low density lipoproteins. In addition to KCs as a source of circulating C1q and ApoE, activated immune cells and particularly macrophages and other cells of myeloid origin are known to produce both C1q and ApoE upon activation (21, 22) at sites of acute or chronic tissue inflammation (23). While the source of the large majority of circulating ApoE is derived from liver hepatocytes (22, 24), myeloid cells may also be a major source of both C1q and ApoE locally as shown by bone marrow transplantation studies in mice (21).

## Materials and methods

### Mice

FVB/NJ WT and FVB.129P2-Abcb4tm1Bor/J were purchased from Charles River Laboratories and housed in the animal facilities of Munich University. A total number of 6 FVB/NJ WT (3 female/3 male) and 6 FVB.129P2-Abcb4tm1Bor/J (3 female/3 male) have been used in this study. Mice were fed a standard rodent chow diet under pathogen free conditions. At 3 month of age liver tissue was dissected and embedded in Tissue-Tec (Sakura Finetek) and stored at -80°C. Animal procedures were approved by the Animal Care and Use Committee of Regierung of Oberbayern.

### Human tissues

All human liver tissues were collected and provided by the biobank of the Technical University of Munich, Klinikum rechts der Isar. Samples were collected within the first 30 minutes after resection. They were macroscopically dissected by an experienced pathologist, snap frozen and stored in liquid nitrogen until further usage. A summary of the human tissues is available in **Supplementary Table 1**. The protocols applied to human samples were approved by the ethics committee of the Faculty of Medicine, Munich University.

### Histology and immunofluorescent microscopy

All stainings were performed on 10 μm fresh-frozen sections. Oil red O (ORO) staining as well as hematoxylin and eosin (HE) staining of liver sections was performed to define liver histopathologies. Immunoflorescent (IF) stainings of mouse liver sections were performed using the following primary antibodies: anti-mouse/human ApoE (ab52607; Abcam); anti-mouse C1q (ab182451; Abcam); anti-mouse CLEC4F (ab2608299; Invitrogen); anti-mouse CD68 (FA11; Serotec); anti-mouse CD31 (ab553370; BD PharMingen); anti-human C3 (A213; ComplementTech); anti-mouse C4 (HM1046; Hycult Biotech); anti-mouse C5 (ab11898, Abcam). We had previously established the specificity of IF microscopy in mouse and human tissues including no primary antibody negative controls, isotype antibody controls, ApoE knockout mouse tissue (i.e., anti-ApoE antibodies) (1). IF stainings of human sections were performed using the following antibodies: anti-mouse/human ApoE (ab52607; Abcam); anti-human C1q (ab71089; Abcam); anti-human CD68 (EMB11; DAKO); anti-human C5 (A220; ComplementTech). Hepatitis sections as well as the appropriate control tissues were fixated with Delaunay solution prior to IF staining. Stained sections were analyzed using a Leica confocal microscope (SP8, Leica, Germany) using Leica Application Suite (Leica) and ImageJ software.

### Proximity ligation assay

Protein-protein interaction *ex vivo* in mouse and human tissues *in situ* were performed using the Duolink^®^ PLA kit (DUO92101 SIGMA) as previously described in (1): briefly, sections were stained with rabbit anti mouse ApoE (ab183597, Abcam) and mouse anti-C1q (HM1096BT, Hycult) for mouse liver tissues and with rabbit anti-human ApoE (ab52607, Abcam) and mouse anti-human C1q (ab71089, Abcam) for human livers. No or only one primary antibody were used as controls. PLA signals were detected according to the manufacturer’s protocol. A Leica confocal microscope (SP8, Leica, Germany) equipped with a 96x or 100x oil objective (NA 1.4) was used for imaging.

### Analyses of single cell transcriptome data

Single cell data analyses were performed using publicly available databases (https://www.livercellatlas.org/download.php) (25). The databases contain 147 samples obtained from mice and human livers with various single cell-based technologies, such as CITE-Seq, Single-nucleus RNA sequencing (Nuc-Seq), and scRNA-Seq. Data were loaded into Rstudio and processed with the Seurat package (version 4.1.0), respectively. The annotation data for each cell was downloaded to define each cell (25). Mice fed with standard diet (SD) were considered healthy controls, mice fed with Western diet (WD): 58% fat, 1% cholesterol, and drinking water with 23.1 g/L fructose and 18.9 g/L sucrose were considered diseased mice (25). Human liver biopsies were collected from patients undergoing cholecystectomy or gastric bypass, and healthy adjacent liver tissue removed during liver resection from colorectal cancer metastasis patients (25). Patients with less than 10% steatosis are regarded as healthy controls; patients with more than 10% steatosis were considered diseased (25, 26). For human studies, a total of 125.844 cells were used, 8.383 hepatocytes (nucSeq) and 1.287 KCs (citeSeq). In mouse studies a total of 56.407 (CD45^+^) and 33.241 (CD45^-^) were used out of which 9.190 were hepatocytes and 5.265 KCs. Violin plots visualized the normalized expression of the selected genes by using *Seurat*. To evaluate the difference of gene expression between different groups, data were analyzed using *Wilcoxon rank sum test* in two groups or *Kruskal-Walli’s test with Dunn’s non-parametric all-pairs comparison test* in multiple groups. The p values were adjusted using *Benjamini Hochberg correction*. * p values <0.05; ** p values <0.01; *** p values <0.001.

## Results

### Cellular expression patterns of C1q and ApoE in Mdr2-KO livers

**Figure 1:**
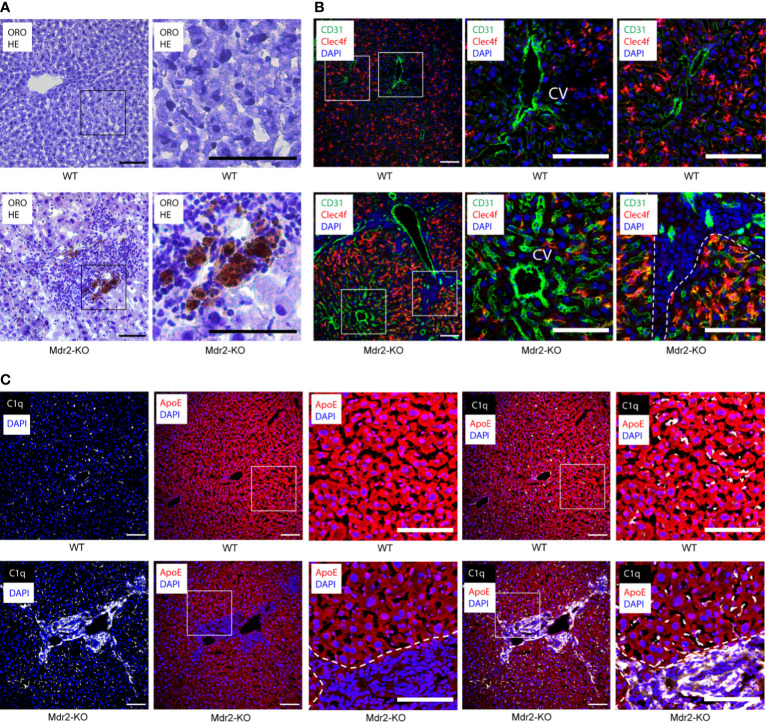
Localization of C1q and ApoE proteins in 3 month old WT and Mdr2-KO mouse livers. **(A)** Liver sections were stained with ORO for lipid (red) and HE for nuclei (blue). In Mdr2-KO immune cell infiltrates form primarily in the portal tract areas as well as in the interlobular space. Lipid droplets were observed within the infiltration sites. Black squares represent high magnification images below. **(B)** Sections were stained with CD31 for vascular structures (green), Clec4f for KCs (red) and DAPI for nuclei (blue). CV stands for central vein. Dotted lines demarcate immune cell infiltrates in the portal tract areas from surrounding liver tissue. An increase of CD31 expression in Mdr2-KO livers suggests ongoing angiogenesis. White squares represent high magnification images shown separately. **(C)** Liver sections were stained with ApoE (red), C1q (white) and DAPI for nuclei (blue). White squares represent high magnification images shown separately. Representative images of WT livers (n=6); Mdr2-KO livers (n=6) are shown. Scale bars 100 μm in images of low and high magnification.

We used Mdr2-KO mice as a widely used model for human chronic hepatitis and HCC pathogenesis (18). A combination of ORO and HE staining at the peak of liver inflammation in Mdr2-KO mice at 3 months of age vs their sex- and age-matched controls, showed patches of inflammatory cells that were associated with lipid deposits. These areas were pronounced in the portal triads in Mdr2-KO liver lobules but not in healthy livers (**Figure 1A**). Antisera against CD31 delineated blood vessels, Clec4f stained KCs, and DAPI indicated nuclei. Radiating from the CD31^+^ central vein (CV) of each lobule towards the portal triads, Clec4f^+^ KCs were located along the sinusoids throughout the concentric centrolobular, midzonal and periportal parts of WT livers (27) as expected (**Figure 1B**). In Mdr2-KO livers, a similar localization for KCs was apparent (**Figure 1B**) but CD31^+^ blood vessels were more abundant and sinusoids were enlarged, distended and more packed indicating neoangiogenesis in the Mdr2-KO lobules (27, 28). We next used antisera to stain C1q and ApoE in livers of WT and Mdr2-KO mice. In WT mice, C1q positivity located along the sinusoids similar to Clec4f but C1q was not observed in hepatocytes (**Figure 1C**), whereas ApoE signals were strong in hepatocytes (**Figure 1C**). In Mdr2-KO mice, C1q positivity was seen along the sinusoids (**Figure 1C**) similar to WT livers. However, unlike WT livers, Mdr2-KO livers revealed very strong C1q signals in the portal triads (**Figure 1C**). These data indicate that portal triad-infiltrating immune cells highly express C1q in Mdr2-KO livers in addition to KCs. In Mdr2-KO livers, ApoE signals were largely restricted to hepatocytes and - to a lesser degree - in cells of the portal triads (**Figure 1C**).

### C1q- and ApoE-expressing cells in murine WT and Mdr2-KO livers

**Figure 2:**
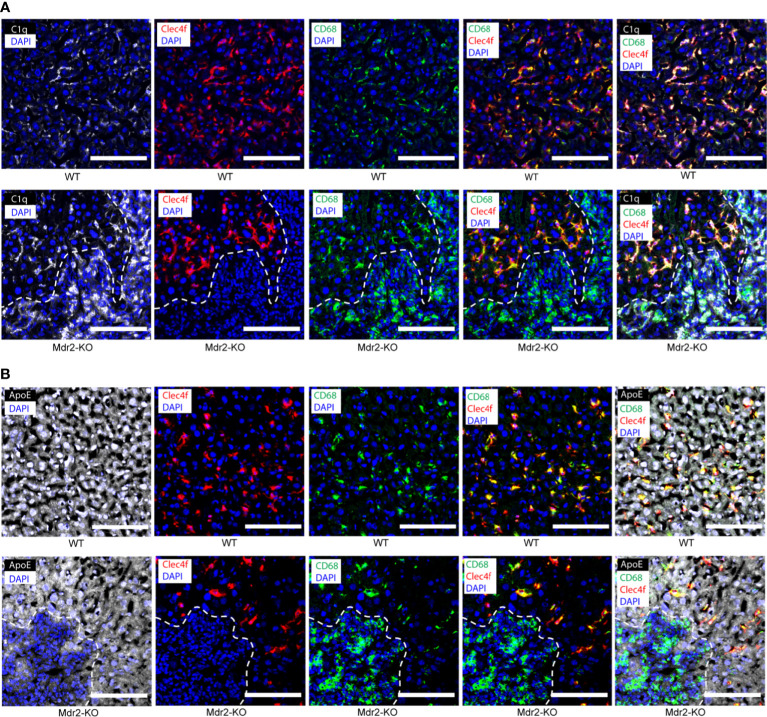
C1q- and ApoE-expressing cells in 3 month old WT and Mdr2-KO mouse livers. **(A)** Liver sections were stained with C1q (white), Clec4f (red) for KCs, CD68 (green) for macrophages and DAPI (blue) for nuclei. Dotted lines demarcate immune cell infiltrates in the portal tract areas from surrounding liver tissue. KCs are known to stain CD68^+^/Clec4f^+^. **(B)** Liver sections were stained with ApoE (white), Clec4f (red) for KCs, CD68 (green) for macrophages and DAPI (blue) for nuclei. Dotted lines demarcate immune cell infiltrates in the portal tract areas from surrounding liver tissue. Representative images of WT livers (n=6); Mdr2-KO livers (n=6) are shown. Scale bars 100 μm.

To distinguish KCs from monocyte-derived macrophages (27), we used Clec4f as a specific marker for mouse KCs together with CD68 as a distinct marker combination for KCs, i.e. KCs stain Clec4f^+^/CD68^+^ whereas macrophages are Clec4f negative but CD68 positive and thus stain Clec4f^-^/CD68^+^. In WT liver sinusoids, most C1q^+^ cells stained double positive for Clec4f and CD68 indicating that KCs are the major C1q-expressing cell type in healthy livers (**Figure 2A**). In addition to KCs, however, Clec4f^-^/CD68^+^ liver parenchymal macrophages were also readily detected but their number was low when compared to KCs though WT liver parenchymal macrophages also stained positive for C1q (**Figure 2A**) indicating that the healthy liver contains two types of C1q-expressing cells, i.e. the majority are KCs and CD68 single positive parenchymal macrophages. In the parenchyma of Mdr2-KO lobules, the staining pattern of C1q in KCs versus parenchymal macrophages was similar to WT livers with most KCs staining positive for Clec4f and CD68 with some single CD68^+^ macrophages also staining positive for C1q (**Figure 2A**). We note, however, that the morphology of KCs in Mdr2-KO mice differed from KCs of WT KCs in that they appeared to have a more elaborate and elongated network of cell extensions (**Figure 2**). Moreover, large macrophage infiltrates were observed in Mdr2-KO livers around portal triads (**Figure 2A**). The portal triad-associated macrophages stained strongly for C1q, indicating that these macrophages are a second major source of C1q protein in Mdr2-KO livers (**Figure 2A**). We next co-stained ApoE with Clec4f and CD68. ApoE was strongly expressed ubiquitously by hepatocytes throughout the lobules both in WT and Mdr2-KO livers (**Figure 2B**). In addition, hepatocytes, Clec4f^+^/CD68^+^ KCs and Clec4f^-^CD68^+^ macrophages in the parenchyma stained positive for ApoE (**Figure 2B**). In Mdr2-KO livers, ApoE was widely expressed by hepatocytes, KCs and parenchymal macrophages in non-inflamed liver parenchyma (**Figure 2B**). However, ApoE stained positive in portal triad macrophages though somewhat weaker compared to hepatocytes (**Figure 2B**).

### Complement components in WT and Mdr2-KO livers

**Figure 3:**
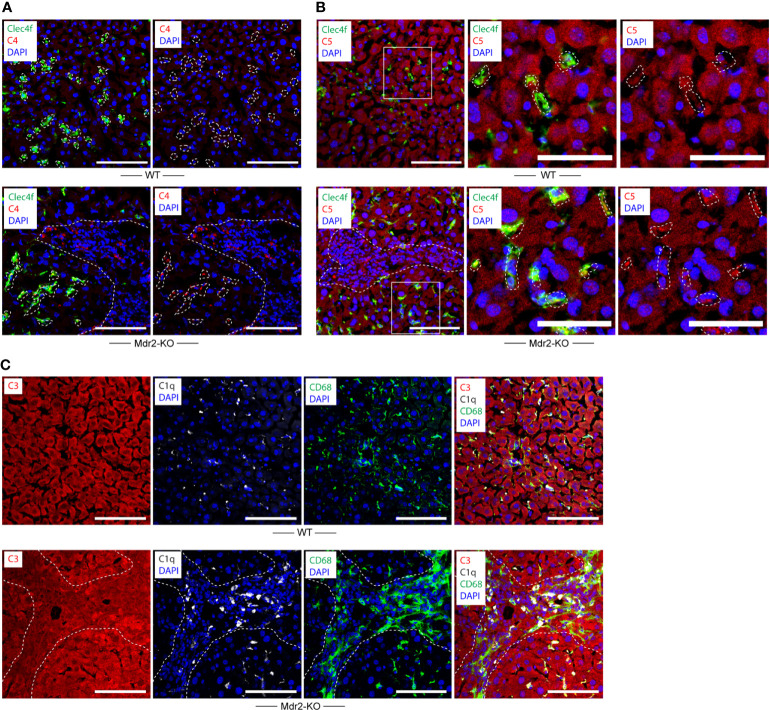
Complement component C4 and C5 expression in WT and Mdr2-KO mouse livers. **(A)** Liver sections were stained with Clec4f (green) for KCs, C4 (red), and DAPI for nuclei (blue). Dotted lines demarcate Clecf^+^ cells as well as immune cell infiltrates in portal triads from adjacent liver parenchyma. **(B)** Liver sections were stained with Clec4f (green) for KCs, C5 (red), and DAPI (blue) for nuclei. **(C)** Complement component C3, C1q, CD68 expression in WT and Mdr2-KO livers. Liver sections were stained with C3 (red), C1q (white), and CD68 (green) for macrophages/KCs and DAPI for nuclei (blue). Dotted lines demarcate immune cell infiltration sites from adjacent liver parenchyma. White squares represent high magnification images shown separately. Representative images of WT livers (n=6); Mdr2-KO livers (n=6) are shown. Scale bars 100 μm in images of low and high magnification.

We next examined complement proteins in WT and Mdr2-KO livers. Complement component C4 was rather weak in WT KCs and hepatocytes (**Figure 3A**) when compared to KCs of Mdr2-KO livers (**Figure 3A**). These data indicated that KCs up-regulate C4 in Mdr2-KO livers. In addition, a subpopulation of Mdr2-KO portal triad macrophages stained strongly positive for C4 (**Figure 3A**). We next examined complement C5. Similar to C4, complement component C5 stained weakly in KCs of WT mice (**Figure 3B**). However, unlike C4, hepatocytes were strongly C5 positive in the WT and Mdr2-KO livers (**Figure 3B**). Similar to C4, C5 appeared to be upregulated in Mdr2-KO KCs and in a subpopulation of portal triad macrophages (**Figure 3B**). We next examined complement component C3 expression in WT and Mdr2-KO mice. C3 protein was highly expressed by hepatocytes in WT mice as well as in Mdr2-KO mice (**Figure 3C**). These data indicate differential expression of C4, C5, and C3 in WT livers and an induction of both C4 and C5 in Mdr2-KO KCs as well as significant expression of C4 and C5 in a subpopulation of portal triad macrophages, while, C3 was widely expressed by hepatocytes in both mice.

### C1q-ApoE complex formation in WT and Mdr2-KO livers

**Figure 4:**
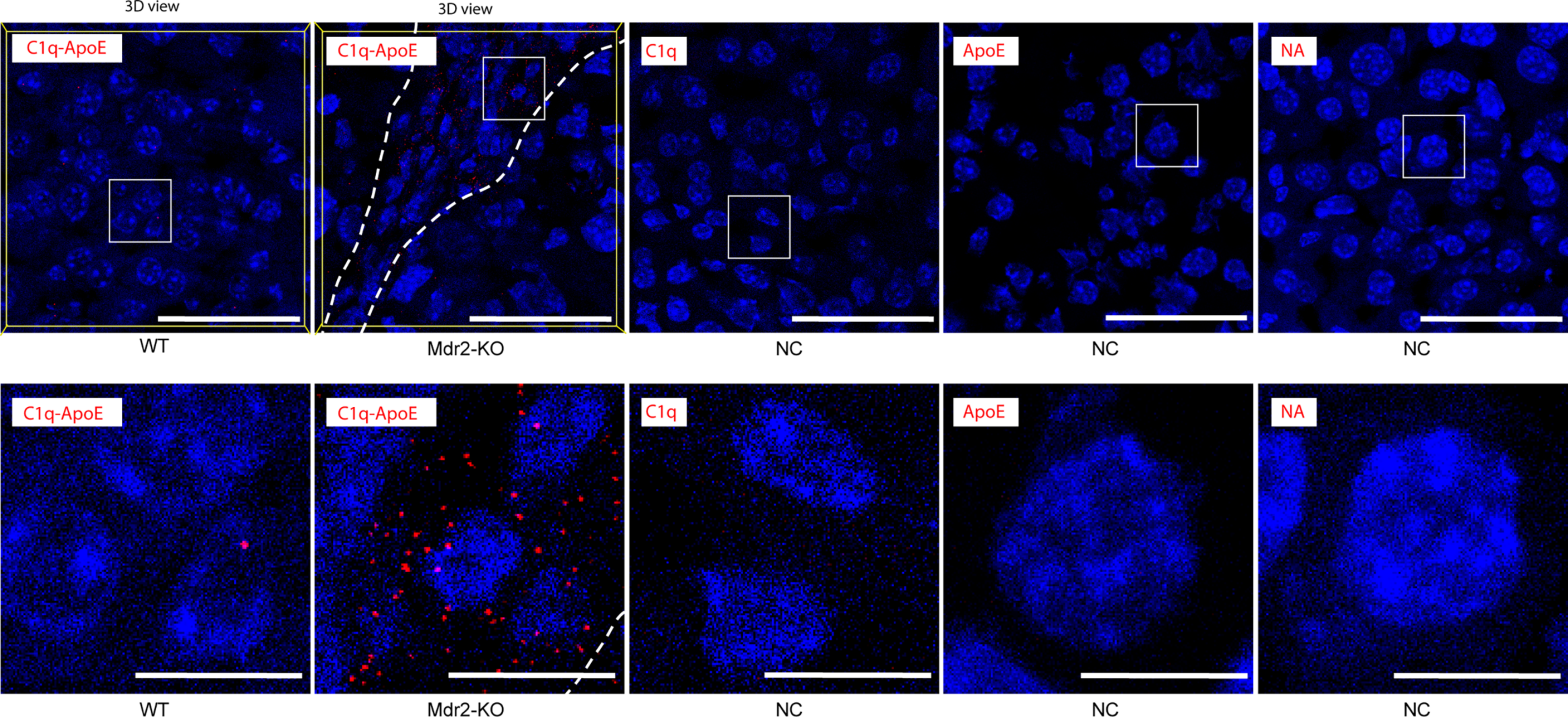
C1q-ApoE complex formation in WT and Mdr2-KO mouse livers. WT mouse liver sections were stained with C1q and ApoE by PLA. Anti-ApoE alone, Anti-C1q alone or no primary antibodies were used as negative controls. Dotted lines demarcate immune cell infiltrations from adjacent liver parenchyma. White square areas represent high magnification images below. 3Dview images are available as mp4 videos in supplements. Representative images of WT livers (n=6); Mdr2-KO livers (n=6) are shown. Scale bar 50 μm in low magnification images and 10 μm in images of high magnification.

To identify the C1q-ApoE complex *in situ*, we used the proximity ligation assay (PLA) as previously described (1). Low levels of C1q-ApoE complexes were observed in WT livers (**Figure 4**, **Supplementary Video 1**). However, C1q-ApoE complexes were readily observed in the portal triads that were infiltrated by immune cells of Mdr2-KO livers (**Figure 4**, **Supplementary Video 2**).

### ScRNAseq analyses of murine healthy and western diet-induced liver steatosis

**Figure 5:**
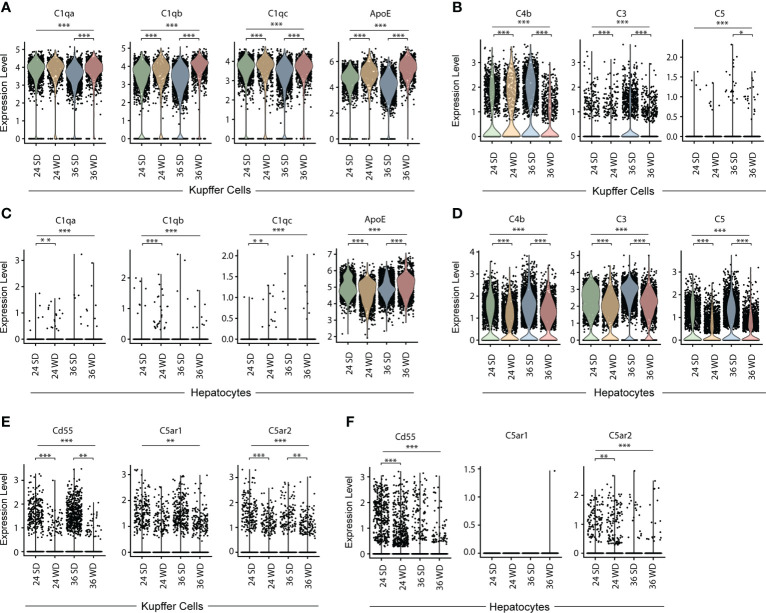
Transcript expression of C1q, ApoE and complement components in KCs and hepatocytes in healthy and diseased mouse livers. **(A, B)** Violin plots show C1qa, C1qb, C1qc, ApoE **(A)** and C4b, C3, and C5 **(B)** transcripts in KCs in standard diet-fed or WD-fed mice for 24 and 36 weeks. **(C, D)** Violin plots show C1qa, C1qb, C1qc, ApoE **(C)** and C4b, C3, and C5 **(D)** transcripts in hepatocytes in healthy and diseased mice. **(E, F)** Violin plots show CD55, C5AR1 and C5AR2 transcripts in KCs **(E)** and hepatocytes **(F)** in healthy and diseased mice. Each point represents one cell. Kruskal–Walli’s test with Dunn’s non-parametric all-pairs comparison test was adjusted by Benjamini Hochberg correction. *P < 0.05; **P < 0.01; ***P < 0.001.

To examine the expression of complement-related transcripts in KCs and hepatocytes in healthy and diseased mice, we mined single cell transcriptomes as reported by others (GSE156059, https://www.livercellatlas.org/download.php) (25, 26). Specifically, C57BL/6 mice fed with a Western diet (WD) high in fat, sucrose and cholesterol for 24 weeks or 36 weeks developed liver pathologies resembling human NAFLD or non-alcoholic steatohepatitis (NASH) (26). Data banks were mined for transcripts of C1qa, C1qb, C1qc, ApoE, C4b, C3, and C5 in KCs of WD-fed mice aged 24 weeks and 36 weeks, as well as their sex- and age-matched healthy control mice. KCs in healthy and diseased mice highly expressed C1qa, C1qb, C1qc, and ApoE transcripts (**Figure 5A**). KCs in diseased mice expressed higher levels of ApoE when compared to healthy controls (**Figure 5B**). We next examined expression of C4b, C3, and C5 in KCs. KCs highly expressed C4b transcripts, but few KCs expressed C3 and C5 transcripts (**Figure 5B**). Hepatocytes in healthy and diseased mice did not express C1q transcripts, but highly expressed ApoE, C4b, C3, and C5 transcripts (**Figures 5C, D**). We next examined the expression of complement receptor CD55, and anaphylatoxin C5a receptors (C5aR1, C5aR2) in KCs and hepatocytes (**Figures 5E, F**). A low percentage of KCs and hepatocytes expressed CD55 and C5aR1/2 transcripts. These data indicate that KCs are the major source of C1q in the liver, and that KCs and hepatocytes are the major source of ApoE in both healthy and diseased livers.

### C1q-ApoE complex formation in human livers with NAFLD and viral hepatitis B and C

**Figure 6:**
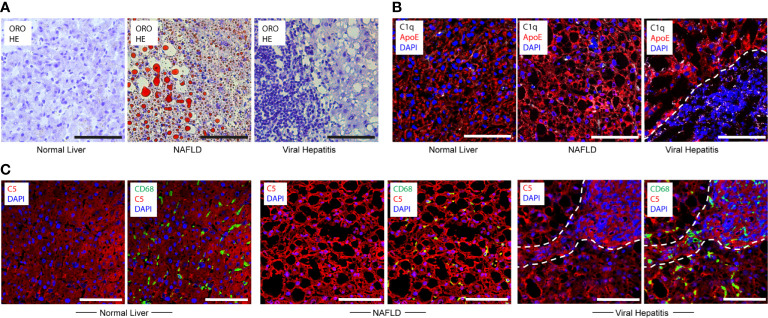
Translational studies in healthy and diseased human livers. **(A)** Normal liver sample Nr. 5 (**Supplementary Table 1**), NAFLD sample Nr. 9 (**Supplementary Table 1**) and viral hepatitis sample Nr. 7 (**Supplementary Table 1**) tissues were stained with ORO for lipid (red) and HE for nuclei (blue). **(B)** Human livers - as described in Figure 6A - were stained with ApoE (red), C1q (white), and DAPI (blue) showing similar staining patterns vs mouse livers. Dotted lines demarcate immune cell infiltration sites from adjacent liver parenchyma. **(C)** Human livers – as described in Figure 6A - were stained with C5 (red), CD68 (green) and DAPI (blue) indicating nuclei. Representative images of human samples (**Supplementary Table 1**) are shown. Dotted lines demarcate immune cell infiltration sites from adjacent liver parenchyma. Scale bars 100 μm.

To examine whether C1q-ApoE complexes form in human livers with acute and chronic inflammatory diseases, we examined human liver biopsies obtained from patients who were diagnosed with NAFLD or hepatitis C and hepatitis B/C coinfection (**Supplementary Table 1**). We stained human livers with ORO for lipid droplets. We did not observe significant lipid droplets or immune cell aggregates in areas of healthy human livers (**Figure 6A**). We noticed intracellular and extracellular lipid droplets of various sizes and apparent immune cells throughout the parenchyma of NAFLD livers as expected (14) (**Figure 6A**). Of note, marked immune cell infiltrates were observed in portal triads whereas lipid droplets in the liver parenchyma in Hep-B/C livers were apparent to a lesser degree (**Figure 6A**). We next examined C1q and ApoE proteins in human livers. ApoE stained positive in all cells in the liver parenchyma and C1q was selectively stained in few cells consistent with KCs in healthy livers (**Figure 6B**). These data indicated that, similar to mouse livers, hepatocytes and KCs stained positive for ApoE, and KCs stained positive for C1q protein in healthy human livers. NAFLD liver parenchyma was interspersed with empty spaces, i.e. lipid droplets. Similar to controls, ApoE positive signals were detected in hepatocytes, and C1q positive signals stained scattered cells, i.e. KCs, in NAFLD livers (**Figure 6B**). In Hep-B/C livers, ApoE stained hepatocytes, C1q stained scattered cells, i.e. KCs in the parenchyma and in infiltration sites (**Figure 6B**). We next stained complement C5 protein in human liver tissues. Complement C5 protein stained positive in healthy-, NAFLD-, and Hep-B/C-liver tissues (**Figure 6C**). These data indicated that complement activation may represent a shared pathology for both NAFLD and viral hepatitis livers.

**Figure 7:**
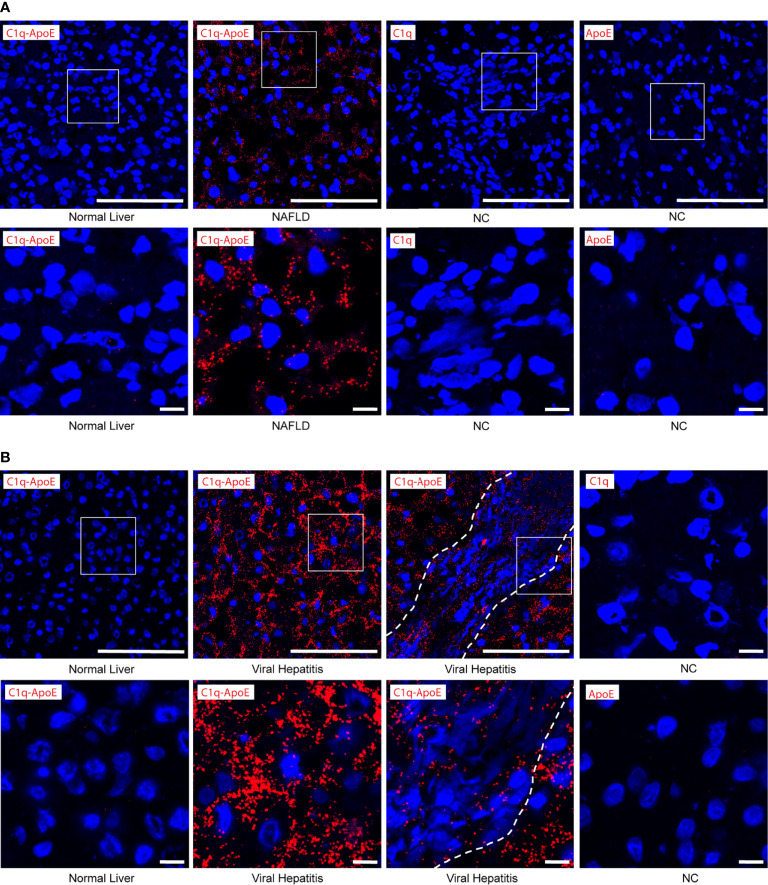
C1q-ApoE complex formation in human livers. **(A, B)** Human liver sections were stained with C1q and ApoE by PLA. Anti-C1q alone or Anti-ApoE alone were used as negative controls. Dotted lines demarcate immune cell infiltrates from adjacent liver parenchyma. White squares represent high magnification images shown separately. Scale bar 100 μm in low magnification images and 10 μm in images of high magnification. Representative images of human samples (**Supplementary Table 1**) are shown. **(A)** Normal liver sample Nr. 2 (**Supplementary Table 1**); NAFLD sample Nr. 9 (**Supplementary Table 1**). **(B)** Normal liver sample Nr. 3 (**Supplementary Table 1**); viral hepatitis sample Nr. 7 (**Supplementary Table 1**).

We next searched for C1q-ApoE complexes *in situ* in human livers using the PLA. No or low levels of C1q-ApoE complexes were observed in control tissues (**Figures 7A, B**). C1q-ApoE complexes were abundantly detected in liver parenchyma obtained from NAFLD patients and in liver parenchyma and in portal infiltration sites of viral hepatitis (**Figures 7A, B**). These data indicated that the C1q-ApoE complex is a new pathological hallmark of NAFLD and viral Hep-B/C-infected livers. The human samples were used as proof-of-principle type experiments to confirm our findings in mice by showing the existence of the C1q-ApoE complex formation within different clinically important human liver diseases. Further studies to be performed in a larger cohort of human livers need to be conducted in future studies including cohorts of sex- and aged matched controls.

**Figure 8:**
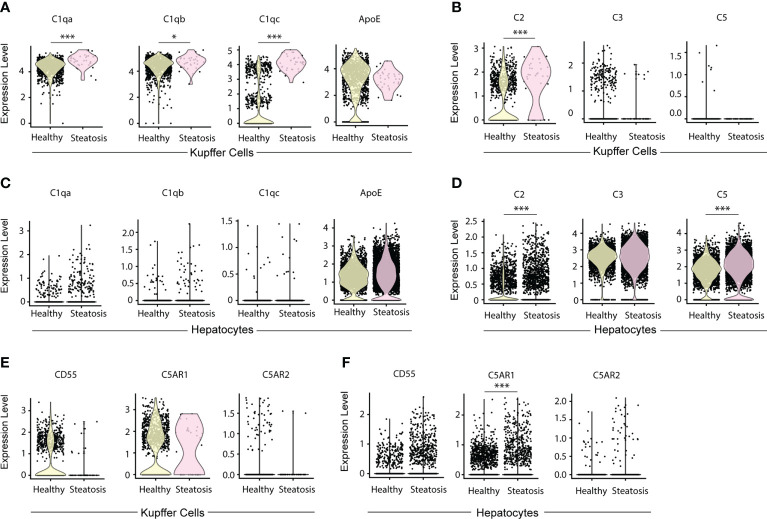
Expression of C1q, ApoE, and complement components in KCs and hepatocytes in human livers. **(A, B)** Violin plots show C1qa, C1qb, C1qc, and ApoE transcripts **(A)** and C2, C3, and C5 transcripts in KCs **(B)** in human livers. **(C, D)** Violin plots show C1qa, C1qb, C1qc, and ApoE transcripts **(C)** and C2, C3, and C5 transcripts in hepatocytes **(D)** in healthy and diseased livers. **(E, F)** Violin plots show CD55, C5AR1 and C5AR2 transcripts in KCs **(E)** and hepatocytes **(F)** in healthy and diseased human livers. Each point represents one cell. Data were analyzed by Wilcoxon rank sum test. P values were adjusted by Benjamini Hochberg correction. *P < 0.05, ***P < 0.001.

To examine complement-related transcript expression in human livers, we searched for C1q, ApoE, C2, C3 and C5 gene expression in published databanks (25). Patients with less than 10% liver steatosis are regarded as healthy controls; patients with more than 10% steatosis were considered diseased (25). KCs in healthy and steatosis patients highly expressed C1qa, C1qb, C1qc, and ApoE transcripts (**Figure 8A**). In healthy human livers there is evidence that specifically C1qc (but not C1qa or C1qb) is differentially expressed in two types of KCs, that i.e. one population with low and another population with high transcript expression per cell (**Figure 8A**). KCs expressed C2 transcripts, but few KCs expressed C3 and C5 transcripts (**Figure 8B**). The expression of C1qa, C1qb, C1qc, and C2 in KCs was significantly higher in patients with severe steatosis when compared to healthy controls (**Figures 8A, B**). Similar to murine hepatocytes, human hepatocytes did not express C1q transcripts (**Figure 8C**) but highly expressed ApoE, C3, and C5 transcripts (**Figures 8C, D**). Similar to mice, these data indicate that KCs are the major source of C1q while KCs and hepatocytes are the major source of ApoE in human livers. We also examined the expression of complement receptor transcript expression in KCs and hepatocytes, including CD55, C5aR1, and C5aR2 (**Figures 8E, F**). The expression of CD55 by KCs is almost undetectable in patients with severe steatosis when compared to healthy controls (**Figure 8E**). The expression of C5aR1 is higher is KCs when compared to hepatocytes, and the expression of C5aR2 is only detectable in few KCs and hepatocytes in both healthy and diseased livers (**Figure 8F**). These data demonstrate in a large number of human versus a large number of mouse single cell transcript analyses of hepatocytes and KCs the striking similarities between mouse and human livers. Moreover, they show that the expression of CCC components as proteins as revealed by IF analyses are analogous. As the mouse versus human transcript versus CCC protein data are comparable, we have also reason to believe that data on a limited number of IF analyses of human livers are tangible.

## Discussion

**Figure 9:**
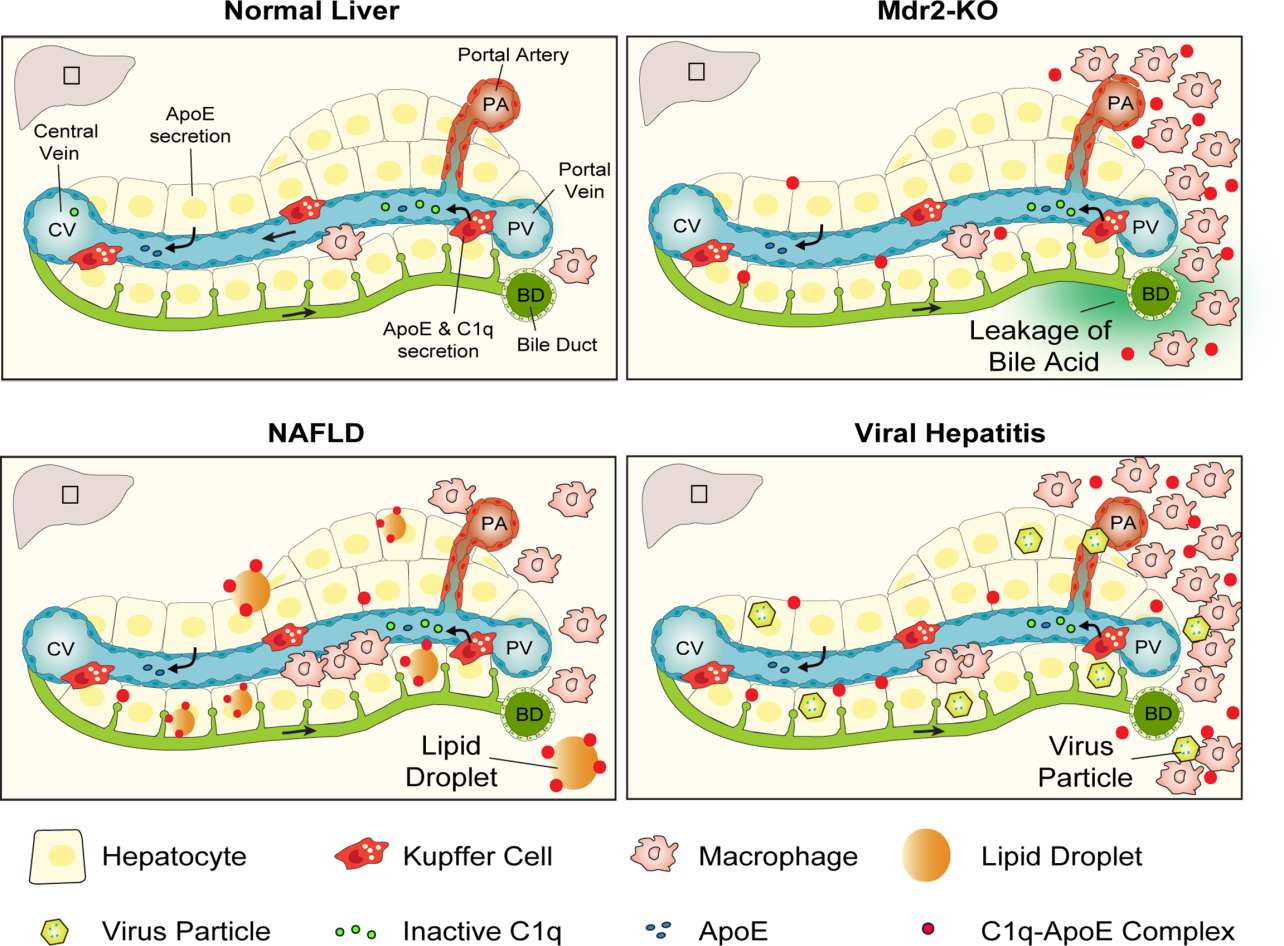
Graphical representation of the C1q-ApoE complex as a new hallmark pathology of diseased liver tissue. In normal liver tissue C1q is secreted into the circulation as an inactive preprotein by KCs as well as monocyte-derived macrophages. ApoE is secreted into the circulation by KCs and hepatocytes. Due to the leakage of bile acid in the portal tract areas of Mdr2-KO mice immune cell infiltrates develop and C1q gets activated and formation of the C1q-ApoE complex is initiated. In NAFLD, C1q is likely to be activated by lipid and in viral hepatitis by virus particles or secondary activators leading to the formation of the C1q-ApoE complex. Schematic Figure modified from (29) with the permission of the authors.

Data reported above support the following conclusions: in healthy murine and human livers the number of C1q-ApoE complexes is low or absent; C1q-ApoE complexes form around portal triads in which activated myeloid cell aggregates emerge and to a lesser degree in the liver parenchyma; C1q-ApoE complexes are abundant in both portal triads and the liver parenchyma in NAFLD livers; numerous C1q-ApoE complexes arise in HepC and HepB/C-infected livers with prominent expression in both portal triads and the liver parenchyma. We conclude that the formation of the C1q-ApoE complex represents a new common pathological hallmark of major forms of human hepatitis including NAFLD (**Figure 9**).

The following sequence of reactions are worth considering: the unique blood flow of the liver combines blood originating from the portal vein and the portal artery with the distinct structure of the liver sinusoids with their KC linings. This results in production and secretion of inactive C1q and ApoE in sinusoidal blood flowing towards the central vein without detectable formation of C1q-ApoE complexes. By contrast, portal inflammation in Mdr2-KO mice, human NAFLD livers and viral hepatitis results in marked formation of the C1q-ApoE complex in portal triads with inflammatory cells. Striking numbers of C1q-ApoE complexes form in both portal triads and the parenchyma of NAFLD and HepB/C-infected livers. These data are consistent with the possibility that there are multiple activators of C1q in the diseased human livers including activators produced in the inflammatory environment of portal triads and extracellular lipid droplets throughout the liver parenchyma. To identify the precise nature of C1q-activating agents in human liver diseases - in particular in NAFLD - requires further work. The purpose of such future studies should correlate the C1q-ApoE complexes with virus particles, immune complexes, lipid droplets, and oxidized lipids as C1q activators may turn out to be new pharmacological targets of CCC activity. These studies may lead to a better understanding of the culprits of C1q activation for potential therapeutic targeting these activators before the CCC is activated with the ultimate goal to prevent liver inflammation as it relates to the CCC. Such strategies may be beneficial particular for patients afflicted with NAFLD and turn out to be effective in the prevention of HCC.

Our observation of the C1q-ApoE complex in acute and chronic hepatitis opens the way for another therapeutic strategy involving ApoE itself. ApoE is a multidomain polymorphic protein with multiple binding sites. The identification of ApoE amino acid sequence that binds to C1q will be important to develop small ApoE peptides that are capable of binding to C1q and concomitantly attenuate its activity to limit detrimental CCC overactivation and HCC.

Several caveats of our study should be considered: The number of clinical samples in this study is rather limited. A clinical study should be performed in which the onset of liver inflammation in NAFLD should be more stringently defined to determine the time course of C1q-ApoE complex formation during subacute NAFLD hepatitis and the various stages of NAFLD including HCC. Furthermore, the C1q-ApoE complex is particularly pronounced in the portal triad where C1q and ApoE seem to be up-regulated in myeloid cells. Indeed, the portal triad-related inflammation may be rich in elusive C1q activators. We further suggest that preclinical studies of fatty liver diseases may yield information whether the course of fatty liver disease is attenuated by pharmaceuticals targeting not only the various components of the three complement pathways but also the C1q-ApoE complex itself and whether liver fibrosis, its development into liver cirrhosis and HCC may be halted by such treatment regimens. ApoE-related replacement therapeutics including ApoE-derived peptides have been developed to treat Alzheimer’s Disease (1, 30, 31). Such approaches may be considered to treat late stages of NAFLD in progressive liver cirrhosis which are associated with major dysfunction of ApoE homeostasis in both the liver and the circulation. Such strategies would be expected to be anti-inflammatory by replacing the bona fide checkpoint inhibitor of activated C1q.

## Data availability statement

Publicly available datasets were analyzed in this study. This data can be found in the GEO repository under accession numbers GSE156059 and GSE192742. All data can be found here: https://www.livercellatlas.org/download.php.

## Ethics statement

The animal study was reviewed and approved by the state of Bavaria, all animal procedures were conducted according to guidelines of the local Animal Use and Care Committees and the National Animal Welfare Laws. The use of human tissue was reviewed and approved by the ethics committee of the Ludwig-Maximilians-University, approval number [21-0095]. Written informed consent for participation was not required for this study in accordance with the national legislation and the institutional requirements.

## Author contributions

LH, ZM and CY designed the experiments, performed experiments, and wrote the manuscript. All authors contributed to the article and approved the submitted version.

## Funding

This study was supported by the Deutsche Forschungsgemeinschaft (DFG) grant YI 133/3-5, and an excellence grant (LMUexcellent 867949-0) of the Ludwig-Maximilians-University Munich to CY.

## Acknowledgments

We thank Shalev Itzkovitz, Department of Cell Biology, Weizmann Institute of Science, Rehovot, Israel, for his permission to use the graphical design of **Figure 1** published in Nat Rev Gastroenterol Hepatol. 2019 Jul; 16(7):395-410.

## Conflict of interest

The authors declare that the research was conducted in the absence of any commercial or financial relationships that could be construed as a potential conflict of interest.

## Publisher’s note

All claims expressed in this article are solely those of the authors and do not necessarily represent those of their affiliated organizations, or those of the publisher, the editors and the reviewers. Any product that may be evaluated in this article, or claim that may be made by its manufacturer, is not guaranteed or endorsed by the publisher.
