# Supplementary material for: The C1q-ApoE complex: A new hallmark pathology of viral hepatitis and nonalcoholic fatty liver disease
Source: Front Immunol. 2022 Oct 6;13:970938. doi: 10.3389/fimmu.2022.970938 (PMC9592549; doi:10.3389/fimmu.2022.970938)
Supplement: Supplementary file 1 [file Table_1.docx]

| **Sample** | **Entity** | **Inflammatory Activity** | **Fatty Degeneration %** | **Fibrosis** | **Age** | **Sex** |
| --- | --- | --- | --- | --- | --- | --- |
| 1 | Normal Liver | 1 | 10 | 0 | 81 | m |
| 2 | Normal Liver | 2 | 15 (max.) | 1 | 45 | f |
| 3 | Normal Liver | 0 | 5 | 1 | 63 | f |
| 4 | Normal Liver | 0-1 | 5 | 0-1 | 46 | m |
| 5 | Normal Liver | 1-2 | 5 | 1-2 | 38 | f |
| 6 | Hepatitis B & C | 3 | 25 | 1 | 61 | m |
| 7 | Hepatitis C | 3 | 20 | 2 | 55 | m |
| 8 | Fatty Liver | 1 | 70 | 2 | 45 | f |
| 9 | Fatty Liver | 1 | 60 | 1 | 60 | m |

**TABLE 1: Characteristics of human liver tissues** Inflammatory Activity: 0-4; Fatty Degeneration in %: Steatosis: >10% <50% of fatty hepatocytes; Fatty Liver: >50% of fatty hepatocytes; Fibrosis: 0-4
